# Beyond Infections: New Warning Signs for Inborn Errors of Immunity in Children

**DOI:** 10.3389/fped.2022.855445

**Published:** 2022-06-10

**Authors:** Giorgio Costagliola, Diego G. Peroni, Rita Consolini

**Affiliations:** ^1^Section of Clinical and Laboratory Immunology, Division of Pediatrics, Department of Clinical and Experimental Medicine, University of Pisa, Pisa, Italy; ^2^Division of Pediatrics, Department of Clinical and Experimental Medicine, University of Pisa, Pisa, Italy

**Keywords:** autoimmune cytopenia, autoimmune lymphoproliferative syndrome, eczema, enteropathy, hyper IgE syndrome (HIES), IPEX syndrome, lymphadenopathy, immunodeficiency–primary

## Abstract

Patients with inborn errors of immunity (IEI) are susceptible to developing a severe infection-related clinical phenotype, but the clinical consequences of immune dysregulation, expressed with autoimmunity, atopy, and lymphoproliferation could represent the first sign in a significant percentage of patients. Therefore, during the diagnostic work-up patients with IEI are frequently addressed to different specialists, including endocrinologists, rheumatologists, and allergologists, often resulting in a delayed diagnosis. In this paper, the most relevant non-infectious manifestations of IEI are discussed. Particularly, we will focus on the potential presentation of IEI with autoimmune cytopenia, non-malignant lymphoproliferation, severe eczema or erythroderma, autoimmune endocrinopathy, enteropathy, and rheumatologic manifestations, including vasculitis and systemic lupus erythematosus. This paper aims to identify new warning signs to suspect IEI and help in the identification of patients presenting with atypical/non-infectious manifestations.

## Introduction

Inborn errors of immunity (IEI) are a wide number of conditions featured by impaired immune response, and their classification is in a continuous update, with the discovery of new clinical entities (1).

**Figure 1:**
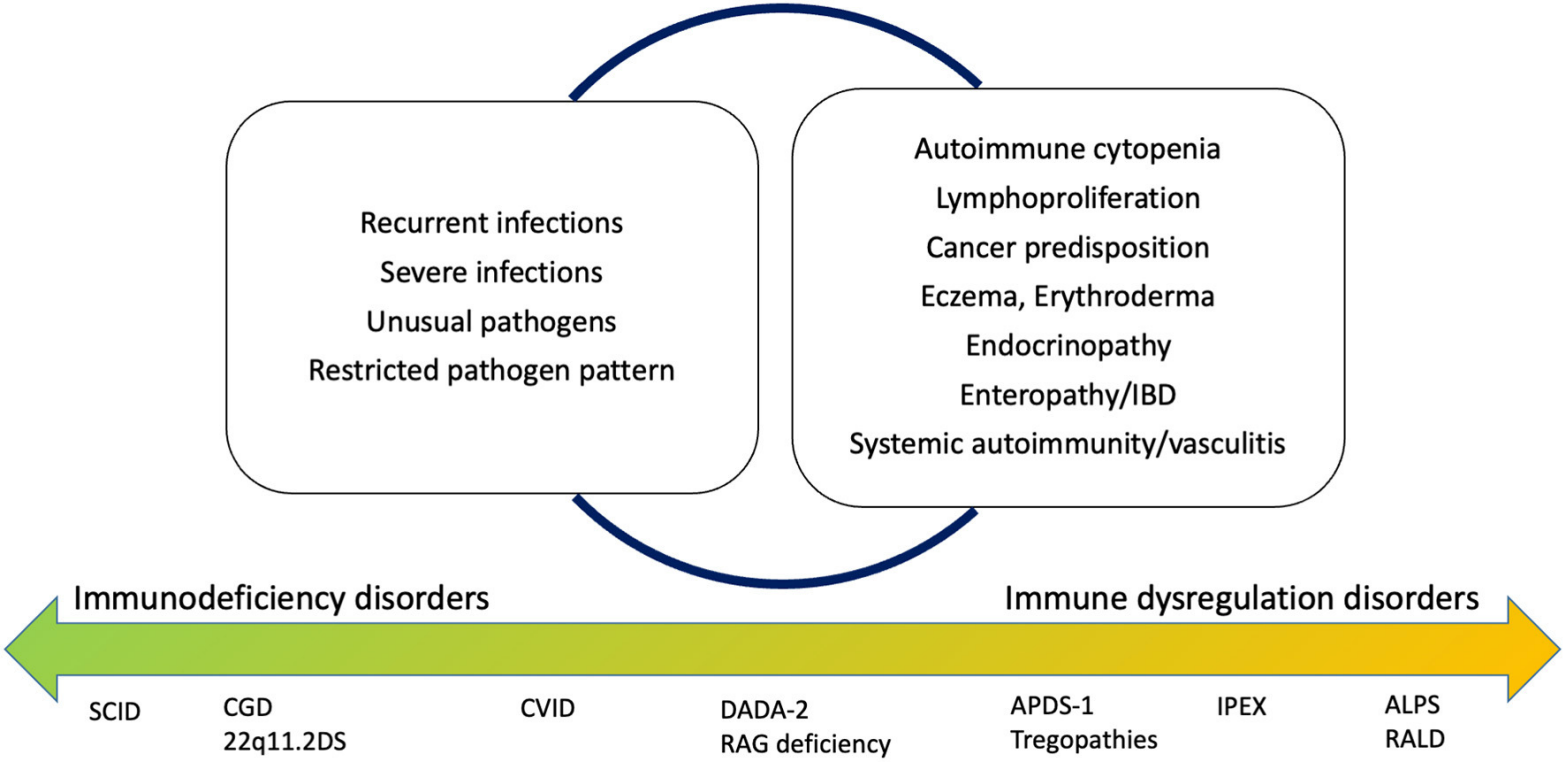
Old and new warning signs for inborn errors of immunity. 22q11.2DS, 22q11.2 deletion syndrome; ALPS, autoimmune lymphoproliferative syndrome; APDS, Activated Phosphoinositide 3-kinase d syndrome; CGD, Chronic granulomatous disease; CVID, Common variable immunodeficiency; DADA-2, deficiency of adenosine deaminase 2; BD, inflammatory bowel disease; IPEX, immune dysregulation, polyendocrinopathy, enteropathy, X-linked stndrome; RALD, RAS-associated leukoproliferative disease; SCID, Severe combined immunodeficiency.

Historically, the warning signs to identify children at risk of IEI have been defined according to the susceptibility to multiple or severe infectious diseases. High recurrence of infections, severe infections with need for hospitalization, use of intravenous antibiotics, and delayed resolution (and recently, infections by unusual pathogens or restricted pathogen susceptibility) are universally recognized as “red flags” for IEI (2). Recent advances in the clinical comprehension of IEI, together with the expansion of their genetic background and the formulation of specific genotype-phenotype correlations, allowed the better characterization of the non-infectious manifestations of IEI (3). Specifically, the immune dysregulation observed in patients with IEI is clinically expressed with autoimmunity, atopy, and lymphoproliferation, and these manifestations represent the first sign of the disease in about 10% of the patients (4, 5). Additionally, new diseases with predominant immune dysregulation phenotype in absence of a significant increase of infections have been identified, leading to an extension of the paradigm of immunodeficiency (6) (Figure 1).

During the diagnostic work-up, patients with non-infectious onset of IEI are frequently addressed to different specialists, including hematologists, endocrinologists, rheumatologists, and allergologists, often resulting in a delayed diagnosis. In this paper, we provide a brief review on the non-infectious manifestations of IEI potentially evidenced at disease onset, focusing on the specific items to be considered for the different clinical specialists. Our work aims to identify new warning signs for the identification of IEI, thus helping in the identification of patients presenting with atypical/non-infectious manifestations.

## Immunodeficiency in the Hematology and Oncology Unit

Several IEI can present with a hematological onset expressed by autoimmune cytopenia or, unexplained benign lymphoproliferation (7). Also, the increasingly recognized role of IEI in hereditary cancer predisposition has a high clinical relevance. Hematologic involvement is described with considerable frequency in patients with common variable immunodeficiency (CVID), combined immunodeficiency disorders (CIDs), and disorders with immune dysregulation, particularly those depending on defective lymphocyte apoptosis of altered regulatory T cell (Treg) function (Tregopathies) (8).

### Autoimmune Cytopenia

Autoimmune cytopenia could represent the first manifestation of both systemic autoimmune diseases and IEI (9). This has been recently quantified in a study by Fisher et al. that demonstrated a 120-fold high risk of developing autoimmune cytopenia in patients with IEI, with a stronger association for autoimmune hemolytic anemia (10). Currently, there are no specific diagnostic biomarkers to identify the patients at risk of IEI among those presenting with autoimmune cytopenia, but unusual age at disease onset (early chronic immune thrombocytopenia, late onset of autoimmune neutropenia), chronic disease course, and refractory disease should be considered as elements of suspect. Therefore, it is reasonable to suggest performing an accurate anamnesis to search relevant or recurrent infections, positive familial history for immune disorders, and analyze serum immunoglobulin levels and the main lymphocyte subpopulation as an initial screening. Moreover, the coexistence of multiple cytopenias should always represent a warning sign for an underlying immune disorder. Concerning this, in a recent study, children with Evans syndromes were tested with a targeted NGS panel including IEI-associated genes, evidencing known mutations in almost half of the children, who tested positive for mutations in CTLA4, LRBA, STAT3, PI3KCD, and others (11). Interestingly, specific immunologic abnormalities (reduced thymic output, increased TCD4+ and TCD8+ central memory compartment) could help in differentiating patients in which autoimmune cytopenia develops in the context of IEI (12).

### Lymphoproliferation

Benign polyclonal lymphoproliferation, clinically expressed by lymphadenopathies and splenomegaly, is also a relevant feature of different IEI (13) (Table 1). Lymphoproliferation is observed in more than 15% of the patients with CVID, with a higher incidence in patients carrying mutations in NF-kB-1 and TACI genes (14). Notably, patients with CVID, and particularly those with other signs of lymphoproliferation, are at higher risk of developing lymphoid malignancies compared to the general population (13), thus highlighting the relevance of formulating a correct diagnosis of CVID in a patient presenting with benign lymphoproliferation. Lymphadenopathies and splenomegaly often represent the leading sign in patients suffering from other IEI, including the activated phosphoinositide 3-kinase delta syndrome (APDS), the autoimmune lymphoproliferative syndrome (ALPS), ALPS-like disorders, Tregopathies, and others (13, 15, 16). APDS is a combined immunodeficiency that could present with a CVID-like phenotype, autoimmune manifestations (mainly cytopenia), and signs of lymphoproliferation, with an increased risk of lymphoid malignancies. In the diagnostic approach to APDS, the increase of senescent T CD8+ cells and transitional B cells plays an important role (15, 17). Differently, in patients suffering from ALPS the infectious phenotype is less relevant, and the diagnostic utility of CD3 double-negative T cells (DNT), the elevation of serum FAS ligand, and vitamin B12 has high predictive value (16, 18). Additionally, it is important to point out that monogenic disorders associated with a defective immune response against Epstein–Barr virus (EBV) present with a clinical picture dominated by the lymphoproliferative features, highlighting the importance of testing for EBV patients with unexplained chronic or recurrent lymphadenopathy and/or splenomegaly (19).

**Table 1:** Non-infectious warning signs for inborn errors of immunity: clinical implications.

**Warning sign**	**Indicators of risk**	**Main associated IEI**	**First-level investigations**	**Second-level investigations: overview**
Autoimmune cytopenia	Multiple cytopenia Refractory disease Chronic course Autoimmune hemolytic anemia	CVID, ALPS, APDS, CTLA4 def, LRBA def, STAT1 GOF, STAT3 GOF	IgA, IgG, IgM Lymphocyte subpopulations with DNTs Vitamin B12	Response to vaccinations Switched memory B cells Senescent T cells Transitional B cells IL-10, IL-18 FAS ligand
Non-malignant lymphoproliferation	Associated cytopenia Familial history of IEI or hematologic malignancy	CVID, ALPS, APDS, CTLA4 def, LRBA def, STAT1 GOF, STAT3 GOF, EBV-related disorders	Blood count (associated cytopenia) IgA, IgG, IgM Lymphocyte subpopulations with DNTs Vitamin B12 EBV PCR (serum and on bioptic specimen if performed)	Response to vaccinations Switched memory B cells Senescent T cells Transitional B cells IL-10, IL-18 FAS ligand
Familial cancer	Familial history of cancer Recurrence of the same neoplasia (myeloid, lymphoid) Personal/familial anamnesis of severe infections or dysregulated immune response	Ataxia-teleangectasia, NBS, Bloom syndrome, Dyskeratosis congenita, GATA2 deficiency	Genetic counseling IgA, IgG, IgM Lymphocyte subpopulations	Bone marrow exam with cytogenetic analysis Testing for germline mutations Lymphocyte proliferation/extended subpopulation analysis on the basis of the specific suspect
Autoimmune endocrinopathies	Multiple endocrine disorders Positive familial history Early disease onset CMC Associated eczema, enteropathy	APS-1, IPEX	Blood count (associated cytopenia, eosinophilia) IgA, IgG, IgM, IgE Lymphocyte subpopulations	Treg cells Autoantibodies (i.e., anti-thyroid, anti-adrenal) if indicated Anti-interferon antibodies
Neonatal hypocalcemia/ hypoparathyroidism	Positive familial history Syndromic features	22q11.2DS	Lymphocyte subpopulations Echocardiography	RTE Naïve T cells Lymphocyte proliferation CGH array if suspect
Eczema	Early onset Severe disease Tooth abnormalities, high palate, increased nasal width, scoliosis History of pneumonia Abscesses Associated endocrinopathy, enteropathy	HIES, IPEX CTLA4 def, LRBA def, STAT1 GOF, STAT3 GOF, WAS	Blood count (eosinophilia, thrombocytopenia) IgA, IgG, IgM, IgE Lymphocyte subpopulations Dermatologic evaluation	Tregs Autoantibodies (i.e., anti-thyroid, anti-adrenal) if indicated Switched memory B cells
Erythroderma	Neonatal onset Chronic disease course Associated lymphoproliferation Syndromic features of 22q11.2DS or CNS (bamboo hair) Associated enteropathy	Omenn syndrome, CNS, 22q11.2DS	TRECs screening Blood count (eosinophilia, lymphopenia) IgA, IgG, IgM Lymphocyte subpopulation Echocardiography Dermatologic evaluation	RTE Naïve T cells Lymphocyte proliferation CGH array if suspect
Pulmonary granulomatous disease (sarcoidosis-like)	Early disease onset Atypical radiologic pattern Extrapulmonary granulomas Absence of other sarcoidosis manifestations	CVID, RAG deficiency, CGD	Blood count (lymphopenia) IgA, IgG, IgM Lymphocyte subpopulations	Response to vaccinations Switched memory B cells Lymphocyte proliferation Granulocyte function (i.e., DHR flow cytometric test)
Early-onset SLE or connective tissue disease	Pre-pubertal onset Male sex Positive familial history Lymphoproliferation	Complement deficiencies Interferonopathies PKCD, RALD	Complement levels IgA, IgG, IgM Lymphocyte subpopulations	Autoantibodies (on the basis of the clinical picture) Interferon signature
Early-onset vasculitis (panarteritis nodosa-like)	Hyperinflammation Lymphoproliferation History of infections	DADA-2	IgA, IgG, IgM Lymphocyte subpopulations	Switched memory B cells Lymphocyte proliferation
Enteropathy	Early onset (first year of life) Positive familial history CMC	IPEX, CTLA4 def, LRBA def, STAT1 GOF, STAT3 GOF	Blood count (associated cytopenia, eosinophilia) IgA, IgG, IgM, IgE Lymphocyte subpopulations	Tregs Autoantibodies (i.e., anti-thyroid, anti-adrenal) if indicated Switched memory B cells
IBD or IBD-like phenotype	Early onset Positive familial history Absence of extraintestinal manifestations (for CGD) Recurrent abscesses	IL-10 pathway deficiency, XLP-2 CGD, Dyskeratosis congenita.	Blood count (associated cytopenia) Liver function IgA, IgG, IgM Lymphocyte subpopulations Abdomen ultrasound	Granulocyte function (i.e., DHR flow cytometric test) Serum cytokines Bone marrow exam if DC or XLP is suspected

*22q11.2DS, 22q11.2 deletion syndrome; ALPS, autoimmune lymphoproliferative syndrome; APDS, Activated Phosphoinositide 3-kinase d syndrome; APS-1, Autoimmune polyendocrine syndrome 1; CGD, Chronic granulomatous disease; CMC, chronic mucocutaneous candidiasis; CNS, Comèl-Netherton syndrome; CVID, Common variable immunodeficiency; DADA-2, deficiency of adenosine deaminase 2; DHR, Dihydrorhodamine; DNTs, Double-negative T cells; HIES, Hyper-IgE syndrome; IBD, inflammatory bowel disease; IPEX, immune dysregulation, polyendocrinopathy, enteropathy, X-linked syndrome; NBS, Nijmegen breakage syndrome; PKCD, protein kinase C δ deficiency; RALD, RAS-associated leukoproliferative disease; RTE, Recent thymic emigrants; WAS, Wiskott-Aldrich syndrome; XLP, X-linked proliferative syndrome*.

### Cancer Susceptibility

Different IEI are associated with a specific genetic predisposition to cancer, that could be the first or only disease presentation. Patients suffering from ataxia-teleangectasia, Bloom syndrome, Nijmegen breakage syndrome, dyskeratosis congenita, and others, show markedly increased risk of cancer, including lymphoid and extra-lymphoid malignancies (20–22). Recently, the expanding genetic background of hereditary cancer syndromes allowed to identify other germline mutations in IEI genes, such as GATA2, associated with combined immunodeficiency and susceptibility to myeloid neoplasms (23), and IKZF1, causing hypogammaglobulinemia and familial lymphoid neoplasms (24). Therefore, although the genetic background of familial cancer syndromes is extremely wide, the possibility of an underlying IEI should be carefully investigated thorough appropriate familial anamnesis and immunological and genetic testing.

## Immunodeficiency in the Endocrinology Unit

Although the association between IEI and endocrine disorders has been extensively studied, when endocrinopathies represent the first or leading sign of a IEI the diagnosis is often difficult. As a general rule, the finding of multiple autoimmune endocrinopathies, the early disease onset, the positive familial history for endocrine disorders and autoimmunity should represent a warning sign for an underlying IEI.

### Autoimmune Endocrinopathies

The paradigm of the immune disorder associated with endocrinopathy is the autoimmune polyendocrine syndrome 1 (APS-1), a rare condition featured by mutations altering the function of the AIRE gene, which has a central role in the process of central negative selection of T lymphocytes and Treg production. Patients with APS-1 present in early life with chronic mucocutaneous candidiasis (CMC) and are prone to the development of different endocrinopathies, mostly Addison's disease and primary hypoparathyroidism. However, patients can also show autoimmune enteropathy, hepatitis, pancreatitis, and nephritis (25). Although there are no specific autoantibodies associated with APS-1, anti-interferon (IFN) antibodies are described in almost all APS-1 patients (8, 26).

Among the disorders associated with multiple endocrinopathies, the immune dysregulation, polyendocrinopathy, enteropathy, X-linked (IPEX) syndrome is of particular interest. In this condition, caused by FOXP3 mutations impairing Treg proliferation and function, patients present with a classic triad of enteropathy, eczematous dermatitis, and endocrinopathies, including thyroiditis and type 1 diabetes (T1D) (27). Blood eosinophilia and increased IgE levels in patients with autoimmune endocrinopathy, especially in case of early onset, should promptly raise the suspect of IPEX (27). Some other Tregopathies without FOXP3 mutations could present with an IPEX-like phenotype. This is the case of patients with CTLA4 deficiency and LRBA deficiency, who present commonly with susceptibility to viral and bacterial infections, lymphoproliferation, and also autoimmunity (28). An IPEX-like phenotype with frequent endocrine involvement is also displayed by patients carrying mutations of the STAT molecular pathways. Particularly, patients with STAT1 gain of function (GOF) mutations show susceptibility to CMC and autoimmune endocrinopathy (mainly thyroiditis and T1D), while the phenotype observed in patients with STAT3 GOF is extremely variable, ranging from IPEX-like presentations to a severe cutaneous and infectious picture (29, 30).

These observations evidence that patients with early-onset or multiple autoimmune endocrinopathies should be accurately investigated for IEI, particularly when there is an association with other features of immune dysregulation. CMC, eczema, enteropathy, or infections, as well as the finding of elevated serum IgE levels and peripheral eosinophilia, should be considered in the diagnostic approach for differential diagnosis.

### Primary Hypoparathyroidism

Another warning relevant sign for IEI that could be evidenced in early life is primary hypoparathyroidism. Indeed, it can be associated with 22q11.2 deletion syndrome (22q11.2DS), although some patients develop only transient neonatal hypocalcemia (31). A reduced thymic output, with low levels of recent thymic emigrants and naïve T cells, together with the observation of the other specific syndromic features (including cardiac malformations, velo-palatal insufficiency) significantly helps in the diagnostic process of 22q11.2DS.

## Immunodeficiency in the Allergy Unit

There is extreme interest in the potential pathogenic links between atopic disorders, autoimmune diseases, and IEI. Severe eczema and newborn erythroderma are the most common presentations of IEI in this clinical context, while severe refractory urticaria can rarely represent the first sign of a IEI (32).

### Eczema

Hyper-IgE syndrome (HIES), a disease caused by STAT3 loss of function (LOF) and DOCK8 deficiency, is the classic example of an IEI that is frequently addressed to allergy units. Indeed, the disease presents with eczema, elevated eosinophil count, and high serum IgE, thus potentially mimicking an atopic disease (32). However, patients with HIES display a wide range of other clinical features, including the increased susceptibility to cutaneous abscesses, CMC, pneumonia, tooth abnormalities, facies with a high palate and increased nasal width, and scoliosis (33). Diagnostic scores analyzing clinical and laboratory parameters associated with the disease are available, and help in the identification of patients at low, intermediate, and high risk of HIES (34).

Apart from HIES, the finding of eczema is common to other inborn errors of immunity, including Wiskott-Aldrich syndrome (WAS), in which it is one of the typical findings together with recurrent infections and thrombocytopenia with low-size platelets (35). Moreover, eczema is part of the classic triad of IPEX, as previously discussed, and is found with considerable frequency in the other Treg-associated (IPEX-like) disorders, particularly STAT3 GOF (29).

The described findings highlight that in a patient with severe, unresponsive eczema, particularly when there is the association with susceptibility to infections, clinicians should be aware of the syndromic features associated with HIES and the manifestations comprised in the clinical spectrum of Tregopathies and WAS. Concerning this, performing a peripheral blood count could significantly help in the diagnostic approach in case of eosinophilia or thrombocytopenia.

### Newborn Erythroderma

Erythroderma could represent the first sign of Omenn syndrome (OS), a subtype of severe combined immunodeficiency featured by the expansion of lymphocytes, peripheral eosinophilia, high serum IgE levels, hepatosplenomegaly, and generalized lymphadenopathy associated with severe, life-threatening infections (36). The high mortality rate of OS in the first month of life, if not adequately treated, highlights the importance of prompt recognition of this disorder. Newborn screening with the demonstration of low levels of T-cell receptor excision circles (TRECS), reduced lymphocyte count, and marked peripheral eosinophilia should be carefully investigated (36, 37). Other than OS, erythroderma could be evidenced in newborns with complete atymia in 22q11.2DS, and in the case of Comèl-Netherton syndrome (CNS), a congenital ichthyosis syndrome caused by a defective skin barrier and clinically associated with erythroderma, bamboo hair, defective antibody response, and enteropathy (38).

Finally, although in rare cases, severe refractory urticaria has been described in association with IEI, potentially representing the first disease manifestation of CVID and a leading sign of the PLCG2-associated antibody deficiency and immune dysregulation (PLAID), thus highlighting the importance of an immunological assessment in selected patients presenting with urticaria (39, 40).

## Immunodeficiency in the Rheumatology Unit

Beyond organ-specific autoimmune disorders, systemic autoimmunity is also evidenced in patients with IEI, and in some peculiar cases the clinical presentation could be misleading. Among the “rheumatologic” manifestations, sarcoidosis-like phenotype, early-onset systemic autoimmunity (particularly, systemic lupus erythematosus [SLE]), and vasculitis could represent warning signs for IEI (41). Positive familial history, association of multiple autoimmune disorders, features of lymphoproliferation, or hyperinflammation represent important elements in the identification of high-risk patients.

### Granulomatous Disease

A paradigmatic condition is represented by IEI with lymphoproliferation and pulmonary involvement, which could manifest in the form of granulomatous lymphocytic interstitial lung disease but also with a sarcoidosis-like clinical picture featured by pulmonary and systemic granulomatous lymphadenopathy. This condition is more common in patients presenting with the granulomatous phenotype of CVID (42, 43), in which immunological assessment can be significantly delayed. A similar clinical phenotype, featured by diffuse granulomatous disease, is also proper of hypomorphic RAG mutations, in which patients display a combined immunodeficiency with variable degree of severity and immune dysregulation (36). Pulmonary, lymphnodal, and extranodal granulomas can also be a presenting feature in patients with the chronic granulomatous disease (CGD), although a clear infectious phenotype with severe and recurrent abscesses represents its most common clinical presentation (44).

### Connective Tissue Diseases and Vasculitis

The case of early-onset connective tissue disease, and particularly SLE, should raise the suspicion of a potentially underlying IEI. Indeed, patients with specific complement deficiencies (C1q, C1r/s, C2, C4a, C4b) show a markedly increased risk for the development of SLE and other autoimmune disorders, including dermatomyositis and juvenile idiopathic arthritis (45). As low complement levels are part of the diagnostic criteria of SLE and are also used as markers of disease activity (46), the identification of a complement deficiency may represent a considerable challenge. Concerning this, the persistence of low complement levels in patients without other clinical or laboratory indicators of active disease should raise the suspicion of an underlying defect. Moreover, given the well-recognized influence of sex hormones in the pathogenesis of connective tissue diseases (47), the pre-puberal onset, as well as the presentation of these disorders in males, can represent a warning sign to search a specific genetic background.

Apart from complement deficiencies, SLE and other connective tissue diseases can develop in patients carrying protein kinase delta deficiency (PKCD), a rare immune disorder with immune dysregulation and lymphoproliferation (48). Similarly, patients with the RAS-associated leukoproliferative disease (RALD) could present with early-onset SLE-like phenotype associated with lymphadenopathy and hepatosplenomegaly (49).

Finally, early-onset SLE can represent the leading manifestation of disorders featured by a dysregulation of the IFN pathway, classified as “interferonopathies,” in which IFN signaling is overactivated, as demonstrated by the analysis of the “IFN signature” (50, 51).

Finally, it is worth to highlight that early-onset systemic vasculitis could rarely reveal disorders of the immune system. Particularly, the deficiency of adenosine deaminase 2 (DADA-2) is a condition that results in a pathogenic crossroad between inflammatory disorders and immunodeficiencies (46), and is featured by polyarteritis nodosa, livedo reticularis, renal involvement, lymphoproliferation, and susceptibility to viral and bacterial infections (52).

## Immunodeficiency in the Gastroenterology Unit

The gastrointestinal system can be involved in a wide range of IEI, including CVID, chronic granulomatous disease, and Tregopathies. Usually, gastrointestinal manifestations are not observed at the initial stage of IEI, and this is particularly relevant for patients with CVID, who commonly develop enteropathy or gastritis in the third or fourth decade of life and are susceptible to develop gastric and intestinal neoplasia, as well as patients with X-linked agammaglobulinemia (XLA) (52). However, some IEI could present to clinical attention only for gastrointestinal involvement. Concerning this, early-onset enteropathy and inflammatory bowel disease (IBD) are the most well-recognized gastrointestinal presentations of IEI (53).

Beyond the definition of new warning signs for IEI, it is worth remembering the well-known association between celiac disease and IgA deficiency, although celiac disease can be observed in several other IEI, including CVID and 22q11.2 DS, highlighting the importance of a first-level immune assessment in children diagnosed with this condition (53–55).

### Enteropathy and Inflammatory Bowel Disease

Early-onset enteropathy (watery and bloody diarrhea with malabsorption), presenting in the first months of life, deserves significant attention. Indeed, it is one of the leading signs of IPEX, often in the context of the classic triad with eczema and endocrinopathy, while in IPEX-like disorders (CTLA-4 deficiency, LRBA deficiency) enteropathy is less frequently a presentation sign and lymphoproliferative or hematologic features can be prominent (27–29, 56).

The association between IBD and IEI is also of interest. Monogenic causes of IBD involving genes implicated in the immune response have been discovered, and account for 10–20% of pediatric IBD patients (56). The risk for an underlying immune disorder is higher for patients with early disease onset, positive familial history for IBD, and extraintestinal manifestations. The most commonly associated genetic conditions are X-linked proliferative syndrome 2 (XLP-2) and IL-10 deficiency, but also XLA, CVID, WAS, and other combined immunodeficiencies have been described (57, 58). Notably, the possibility to present with IBD as a first sign (potentially in the first year of life) is proper of defects of the IL-10 molecular pathway and defects influencing the gut epithelial barrier, such as the dyskeratosis congenita (57, 58).

Also, gastrointestinal involvement is a common finding in patients with CGD in the form of granulomatous colitis, which can present with a clinical and histological phenotype mimicking Crohn's disease (CD) but lacking the typical extraintestinal manifestations of IBD (57, 59). Usually, CGD-associated colitis has an earlier onset compared to CD, and is often accompanied by a positive anamnesis for recurrent or severe abscesses in different organs (cutaneous, hepatic, lymphnodal) and delayed umbilical cord scarring (44, 60). Concerning this, patients with early-onset CD, high-risk anamnesis, and positive familial history should undergo an immunological assessment with the evaluation of phagocytic function and, when appropriate, genetic testing for NADPH mutations.

### Hepatic Involvement

Hepatic involvement is reported with considerable frequency in IEI, in the form of hepatomegaly or as the result of autoimmune hepatitis, although hepatic manifestations are rarely the only finding of an IEI at disease onset (61). Interestingly, IEI associated with susceptibility to hemophagocytic lymphoistiocytosis (XLP-1, XLP-2, and others) could present with unexplained pictures of hypertransaminasemia and hyperinflammation, mimicking infectious or autoimmune hepatitis (62); in these patients, recognizing the other HLH-associated clinical and laboratory signs (fever, splenomegaly, cytopenia, elevated serum ferritin and triglycerides, low fibrinogen) is of extreme diagnostic relevance.

## Conclusion

In this work, we provided a brief overview of the main clinical and laboratory signs that could be considered as new, adjunctive warning signs to suspect a IEI, and implement the historical red flags. With a better knowledge of the new warning signs, hopefully, patients presenting with non-infectious features could be accurately interrogated for other IEI-associated manifestations (infectious and non-infectious) and undergo immunological assessment, leading to an earlier diagnosis.

## Author Contributions

GC and RC conceptualized the paper. GC drafted the paper. DP and RC critically revised it. All authors contributed to the article and approved the submitted version.

## Conflict of Interest

The authors declare that the research was conducted in the absence of any commercial or financial relationships that could be construed as a potential conflict of interest.

## Publisher's Note

All claims expressed in this article are solely those of the authors and do not necessarily represent those of their affiliated organizations, or those of the publisher, the editors and the reviewers. Any product that may be evaluated in this article, or claim that may be made by its manufacturer, is not guaranteed or endorsed by the publisher.
